# Identification of MIR600HG/hsa-miR-342-3p/ANLN network as a potential prognosis biomarker associated with lmmune infiltrates in pancreatic cancer

**DOI:** 10.1038/s41598-023-43174-y

**Published:** 2023-09-23

**Authors:** Baolin Qian, Qi Liu, Chaoqun Wang, Shounan Lu, Shanjia Ke, Bing Yin, Xinglong Li, Hongjun Yu, Yaohua Wu, Yong Ma

**Affiliations:** 1 Department of Minimally Invasive Hepatic Surgery, The First Affiliated Hospital of Harbin Medical University, Harbin, China; 2 Key Laboratory of Hepatosplenic Surgery, Ministry of Education, The First Affiliated Hospital of Harbin Medical University, Harbin, China; 3 Department of Pathology, The First Affiliated Hospital of Harbin Medical University, Harbin, China; 4 Department of Thyroid Surgery, The First Affiliated Hospital of Harbin Medical University, Harbin, China

**Keywords:** Gene regulatory networks, Pancreatic cancer

## Abstract

Pancreatic cancer is one of the tumors with the worst prognosis, causing serious harm to human health. The RNA network and immune response play an important role in tumor progression. While a systematic RNA network linked to the tumor immune response remains to be further explored in pancreatic cancer. Based on The Cancer Genome Atlas (TCGA) and Gene Expression Omnibus (GEO) databases, the MIR600HG/hsa-miR-342-3p/ANLN network was determined. WB and IHC were used to confirm the high expression of ANLN in pancreatic cancer. The prognostic model based on the RNA network could effectively predict the survival prognosis of patients. The analysis of immune infiltration showed that the MIR600HG/hsa-miR-342-3p/ANLN network altered the level of infiltration of T helper 2 (Th2) and effector memory T (Tem) cells. Furthermore, we found that the chemokines chemokine ligand (CCL) 5 and CCL14 may play a key role in immune cell infiltration mediated by the RNA network. In conclusion, this study constructed a prognostic model based on the MIR600HG/hsa-miR-342-3p/ANLN network and found that it may function in tumor immunity.

## Introduction

Pancreatic cancer has the worst prognosis among common gastrointestinal malignancies, with a 5-year overall survival (OS) rate of approximately 10%^1^. Pancreatic cancer is characterized by atypical early symptoms and a high degree of malignancy, and surgery is still the most effective treatment for pancreatic cancer^2^. However, most patients with pancreatic cancer are at an advanced stage when they are diagnosed, and miss the opportunity for surgery. Even after surgical resection, they are prone to recurrence and metastasis^3^. Molecular targeted therapy refers to the use of drugs or other substances that designed for specific carcinogenic sites (which can be protein molecules or gene fragments in tumor cells) to inhibit tumor growth or cause tumor cell death without affecting the surrounding normal tissue^4^. In recent years, molecular targeted therapy has developed rapidly, but there are also many shortcomings such as drug resistance and individual differences^5^. In addition, it was reported that women had longer OS compared to men with pancreatic cancer^6^, and cox multivariate analysis suggested that gender was an independent predictor of OS^7^. To date, the exact mechanism of the occurrence and development of pancreatic cancer remains unclear. Thus, investigations into the molecular mechanism of pancreatic cancer and the development of effective therapeutic targets and new prognostic biomarkers for the treatment of pancreatic cancer are very important.

With the proposal of the long noncoding RNA (lncRNA)‒microRNA (miRNA)-messenger (mRNA) network^8^, an increasing number of scholars have begun to pay attention to the effects of mRNAs and noncoding RNAs (mainly including miRNAs and lncRNAs) on the occurrence, development, and prognosis of tumors and the relationship among them. The lncRNA‒miRNA–mRNA network has been confirmed to play an important role in the progression and metastasis of a variety of cancers, such as colorectal cancer^9^, liver cancer^10,11^, and breast cancer^12^. To date, studies have assessed the lncRNA‒miRNA-mRNA network in pancreatic cancer, but most of them are network axes related to the prognosis^13,14^. The key lncRNA‒miRNA–mRNA network related to immune cell infiltration and the prognosis of pancreatic cancer requires further study.

In recent years, with the in-depth understanding of the tumor microenvironment (TME), new insights into the pathogenesis and targeted therapy of pancreatic cancer have been provided. The TME is a mixture of immune cells, stromal cells, extracellular matrix molecules, cytokines and chemokines^15^. The TME of pancreatic cancer is a relatively complex internal environment that is conducive to the growth of tumor cells. One of its notable features is the presence of a large number of dense matrix components, including fibroblasts, blood vessels, pancreatic stellate cells (PSCs) and other substances^16^. These dense matrix components provide favorable conditions for the growth of pancreatic cancer cells. In addition, a variety of immune cells with different functions are present in the TME of pancreatic cancer, such as CD4 + /CD8 + effector T cells, natural killer cells (NK cells) and dendritic cells (DCs), with antitumor effects^17^. These immune cells are less abundant in the TME, while the immunosuppressive regulatory T cells (Tregs), myeloid-derived suppressor cells (MDSCs) and tumor-associated macrophages (TAMs) are present in large numbers in the TME. Their functions are active, and they secrete large amounts of interleukin-10 (IL-10), TGF-β, IDO and other immunosuppressive factors to form an immunosuppressive TME in pancreatic cancer, which inhibits the immune response and causes immune escape to alter the effect of immunotherapy^18^.

In this study, we identified the hub differentially expressed genes (DEGs) in pancreatic cancer by analyzing three Gene Expression Omnibus (GEO) datasets and one dataset from The Cancer Genome Atlas (TCGA), and we verified in patient samples. Immediately, the upstream miRNAs and lncRNAs were predicted, and miRNAs and lncRNAs related to immune cell infiltration and the prognosis of pancreatic cancer were screened in a subsequent analysis. Then, the lncRNA‒miRNA–mRNA regulatory network was established according to their corresponding relationship (Fig. S1). Finally, the miR-600HG-hsa-miR-342-3p-ANLN network was created after screening and refining the data. This regulatory axis may be further studied as a biomarker or drug therapeutic target for pancreatic cancer and provide a new theoretical basis and potential target for the treatment of pancreatic cancer.

## Results

### Screening of key genes

**Figure 1:**
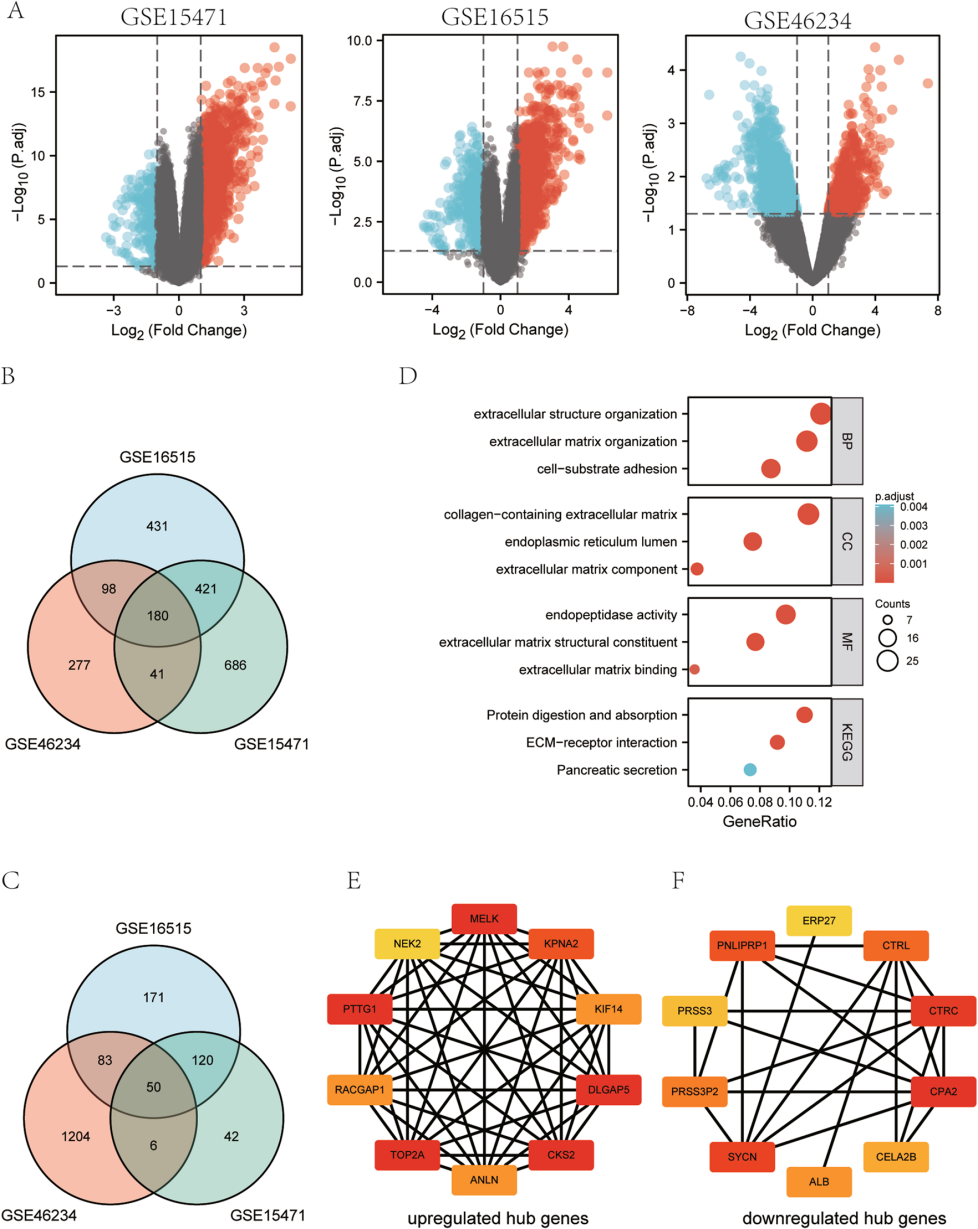
Screening of differentially expressed mRNAs. (**A**) Volcano plots of differentially expressed mRNA. Venn diagram of DEGs in the three gene datasets. (**B**) Upregulated DEGs and (**C**) downregulated DEGs. (**D**) GO and KEGG pathway enrichment analyses of DEGs, including biological process (BP), molecular function (MF), and cellular component (CC). The PPI network and Cytoscape were used to screen hub genes. (**E**) The significantly upregulated genes and (**F**) the significantly downregulated genes.

We analyzed the microarray data (GSE15471, GSE16515, and GSE46234) from patients with pancreatic cancer in the GEO database, and 230 DEGs were obtained (Fig. 1A), including 180 upregulated genes (Fig. 1B) and 50 downregulated genes (Fig. 1C). The enrichment analysis of all DEGs is shown in Fig. 1D. The BP included extracellular structure organization, extracellular matrix organization, and cell-substrate adhesion. The CC included collagen-containing extracellular matrix, endoplasmic reticulum lumen, and extracellular matrix components. The MF consisted of endopeptidase activity, extracellular matrix structural constituent, and extracellular matrix binding. The enrichment analysis of upregulated/downregulated DEGs is shown in Fig. S2A–B. We entered DEGs into the online database STRING to obtain the PPI network and then imported the data into Cytoscape for a visual analysis. Subsequently, 10 upregulated hub genes (Fig. 1E) and 10 downregulated hub genes (Fig. 1F) were selected.

Furthermore, we performed a K-M survival analysis of 20 hub genes based on TCGA database. The results indicated that 10 upregulated hub genes (ANLN, CKS2, DLGAP5, KIF14, KPNA2, MELK, NEK2, PTTG1, RACGAP1, and TOP2A) were related to OS, while only ERP27 (downregulated hub genes) was associated with OS (*P* < 0.05) (Fig. S3). Therefore, this study focused on the 11 key genes in the follow-up analysis. We also verified the expression levels of key genes. Compared with normal tissues, the expression of ANLN, CKS2, DLGAP5, KIF14, KPNA2, MELK, NEK2, PTTG1, RACGAP1, and TOP2A was upregulated, while ERP27 expression was downregulated in pancreatic cancer tissues (*P* < 0.05) (Fig. S4).

### Screening of key miRNAs

**Figure 2:**
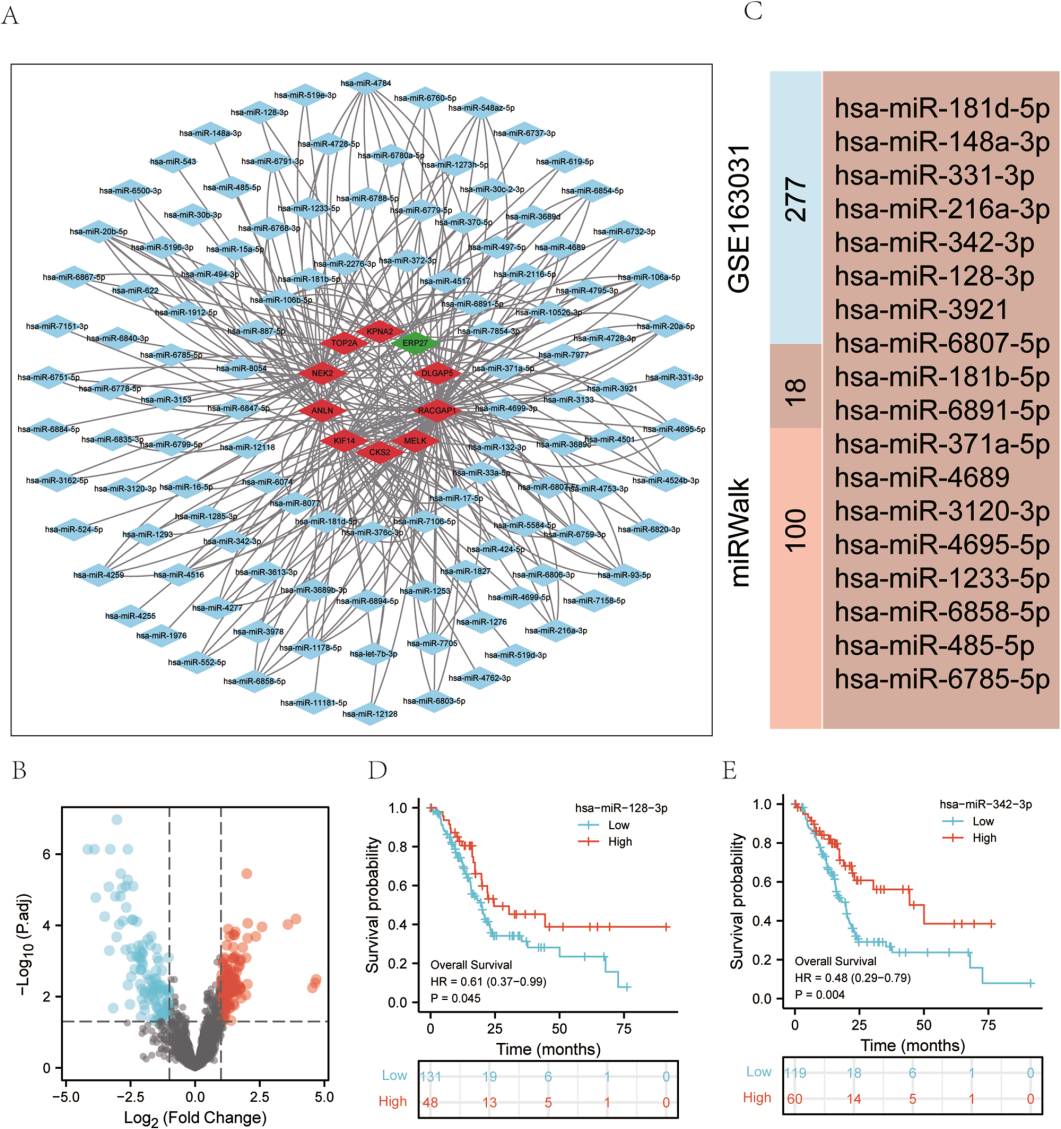
Screening of upstream differentially expressed miRNAs. (**A**) Upstream miRNAs were searched and mRNA‒miRNA networks were constructed. Red indicates upregulated key genes, green indicates downregulated key genes, and blue represent miRNAs. (**B**) Volcano plot of differentially expressed miRNAs. (**C**) Venn diagram of miRNAs between upstream miRNAs (predicted by miRWalk) and differentially expressed miRNAs (screening of GSE163031). (**D**–**E**) The OS analysis based on miRNAs.

To identify the potential key miRNAs, we predicted the upstream miRNAs of key genes by using the miRwalk database, and 118 upstream miRNAs were found. The PPI network visualizing upstream miRNAs is shown in Fig. 2A. Due to of the tissue-specific expression of miRNAs, so we aimed to find the miRNAs that are differentially expressed in pancreatic cancer. Then, 2560 differentially expressed miRNAs between pancreatic cancer and normal tissues were identified in the GSE163031 dataset from the GEO database. The volcano plot showed 295 significantly altered miRNAs (including 153 upregulated miRNAs and 142 downregulated miRNAs) (|logFC|> 1 & p.adj < 0.05) (Fig. 2B). Next, we acquired 18 common miRNAs between the predicted upstream miRNAs and differentially expressed miRNAs in GSE163031 (Fig. 2C). We analyzed the correlation between the miRNAs and the targeted mRNAs. The results indicated that 8 pairs of miRNAs‒mRNAs were significant (*P* < 0.05) (Table S1). Subsequently, the results of the K–M survival analysis suggested that hsa-miR-128-3p and hsa-miR-342-3p were associated with the OS of patients with pancreatic cancer (*P* < 0.05) (Fig. 2D–E). Therefore, we focused on hsa-miR-128-3p and hsa-miR-342-3p in the subsequent analysis.

### Screening of IncRNAs and construction of the RNA network

**Figure 3:**
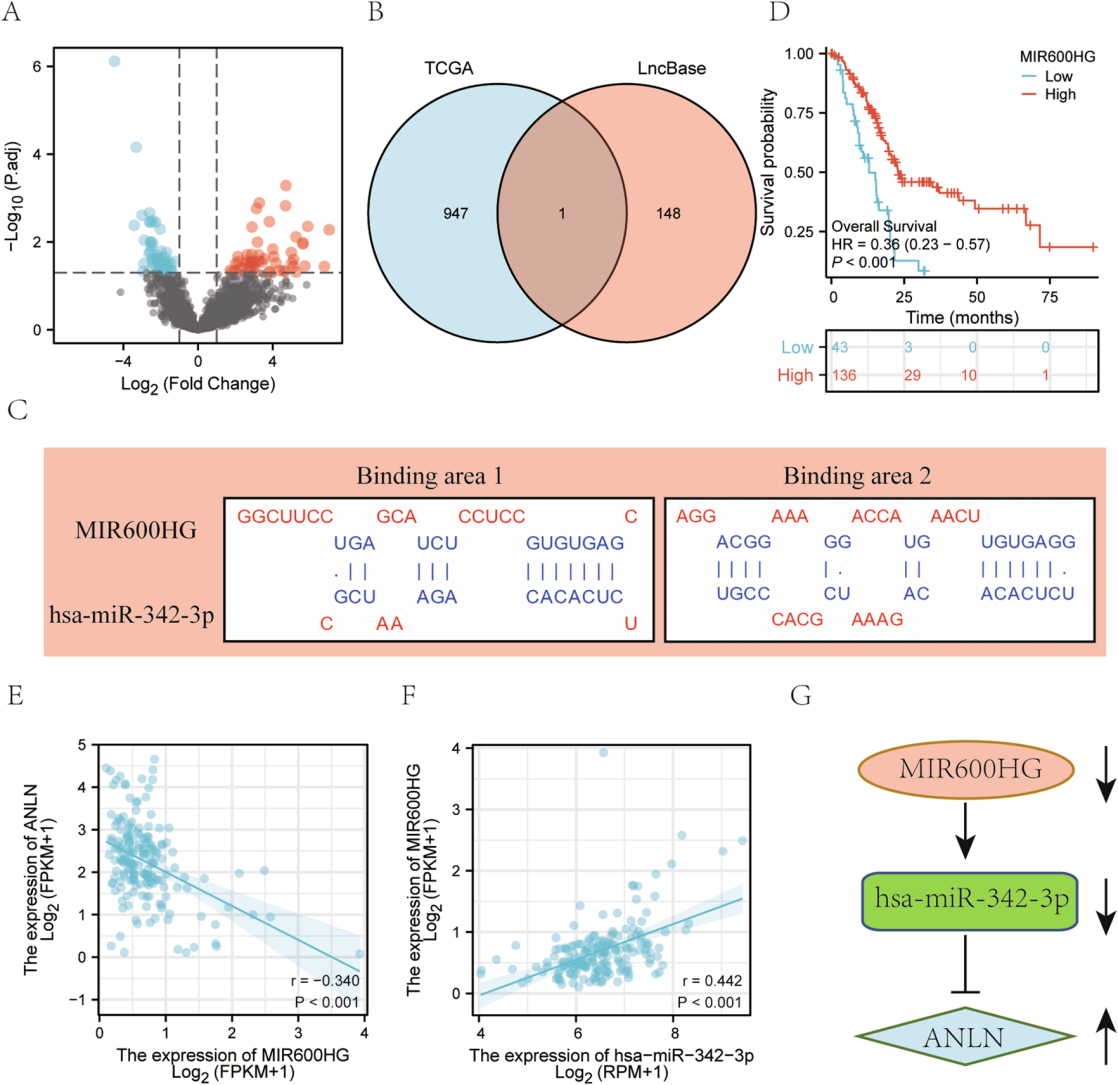
Screening of upstream differentially expressed lncRNA. (**A**) Volcano plot of differentially expressed lncRNAs in pancreatic cancer from TCGA. (**B**) Venn diagram of lncRNAs between upstream lncRNAs (predicted by lncBase) and differentially expressed lncRNAs (screening of TCGA database). (**C**) Base pairing between MIR600HG and hsa-miR-342-3p and the target site predicted by lncBase. (**D**) The Kaplan‒Meier survival curve of the lncRNAs. (**E**) The coexpression of the mRNA (ANLN) and lncRNA (MIR600HG). (**F**) The coexpression of the miRNA (hsa-miR-342-3p) and lncRNA (MIR600HG). (**G**) Diagram of the RNA network.

An increasing number of studies have suggested that lncRNAs function as molecules regulating miRNAs; therefore, we speculated whether a similar regulatory mode also exists for key miRNAs. Here, 5444 differentially expressed lncRNAs between pancreatic cancer and normal tissues were identified in TCGA database. The volcano plot showed 948 significantly differentially expressed lncRNAs (including 473 upregulated lncRNAs and 475 downregulated lncRNAs) (|logFC|> 1 & p.adj < 0.05) (Fig. 3A). In addition, 149 upstream lncRNAs of hsa-miR-128-3p and hsa-miR-342-3p were predicted using the lncBase online website. Then, we acquired the common lncRNA (MIR600HG) between the upstream lncRNAs and significantly differentially expressed lncRNAs from TCGA (Fig. 3B). Subsequently, we analyzed the binding sites between the miRNA (hsa-miR-342-3p) and lncRNA (MIR600HG) using the lncBase online website. As a result, two binding sites for MIR600HG and hsa-miR-342-3p were identified (Fig. 3C). Hence, this study focused on MIR600HG in the follow-up analysis.

According to the K-M survival analysis, MIR600HG was associated with a shorter OS of patients with pancreatic cancer (*P* < 0.05) (Fig. 3D). Furthermore, the results of Pearson’s correlation analysis showed that MIR600HG was negatively correlated with ANLN but positively correlated with hsa-miR-342-3p (Fig. 3E–F). Finally, we obtained a new RNA network (MIR600HG/hsa-miR-342-3p/ANLN) related to the prognosis of patients with pancreatic cancer (Fig. 3G).

### Construction of a MIR600HG/hsa-miR-342-3p/ANLN related risk model

**Figure 4:**
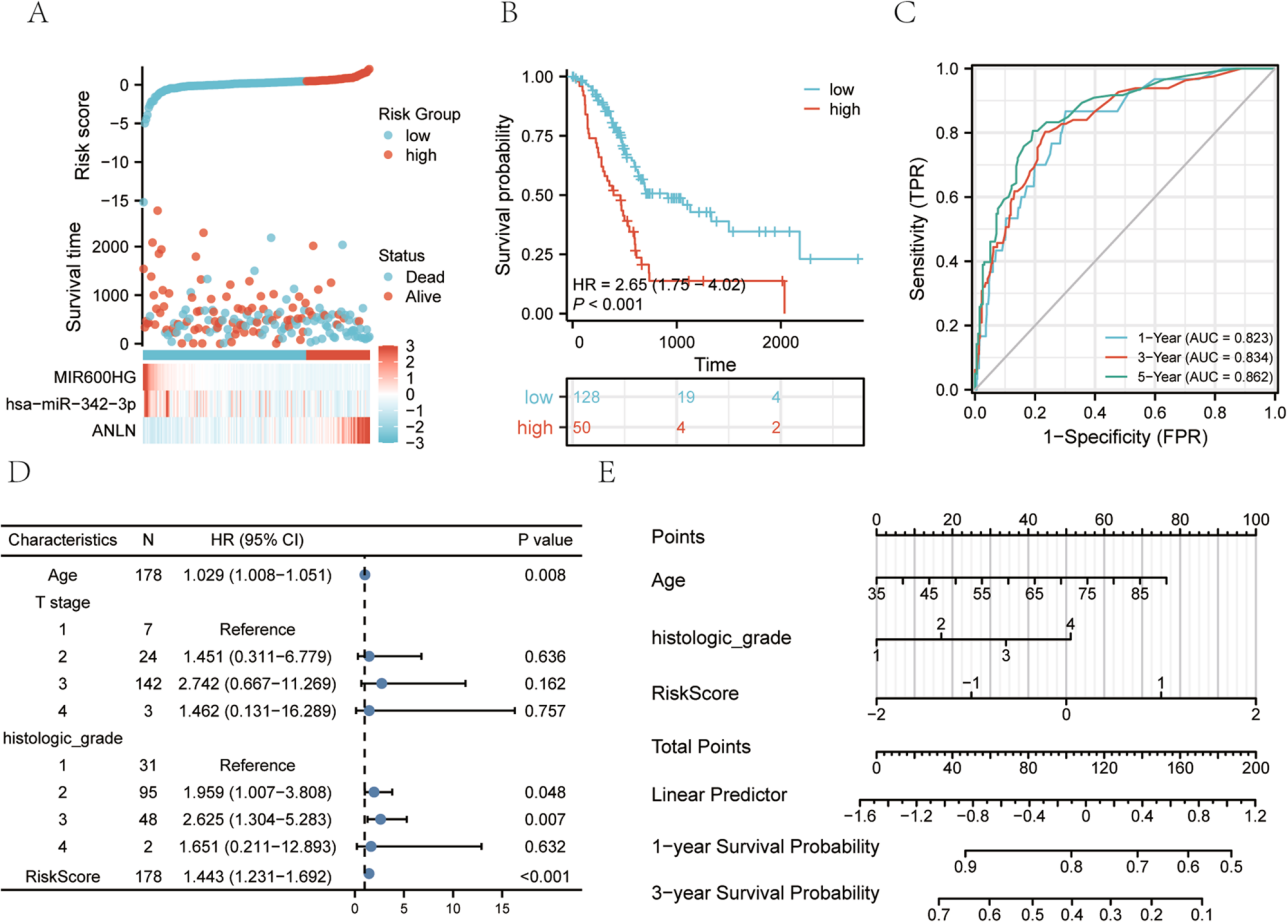
The prognostic model constructed based on the ANLN/hsa-miR-342-3p/MIR600HG network. (**A**) Risk plot for the patients with pancreatic cancer from TCGA. The upper panel shows the risk score and distribution of patients. The middle panel shows the corresponding survival status and survival time of patients. The lower panel shows the expression of ANLN/hsa-miR-342-3p/MIR600HG. (**B**) K–M curve for the risk score based on TCGA. (**C**) Multivariate Cox analysis of independent prognostic risk factors. (**D**) The new prognostic model for predicting the 1-year, 3-year, and 5-year OS of patients with pancreatic cancer based on the RNA network. (**E**) Time-dependent ROC curve of the risk prognostic model. AUC: area under the curve, FPR: false positive rate, and TPR: true positive rate.

From TCGA database, we obtained the clinical characteristics of patients expressing the genes in the network (MIR600HG, hsa-miR-342-3p, and ANLN), as shown in Table S2. We explored whether the MIR600HG/hsa-miR-342-3p/ANLN network could be used as an indicator to evaluate the prognosis of patients with pancreatic cancer by investigating the association of the RNA network and OS in patients with pancreatic cancer using a multivariate Cox regression analysis. Patients with pancreatic cancer were divided into a high-risk subgroup and a low-risk subgroup according to the median value of the RNA network risk score (Fig. 4A). By performing a K–M survival analysis, we found that patients with pancreatic cancer presenting high risk scores had a poor clinical prognosis (Fig. 4B).

It is well known that the prognosis of patients with pancreatic cancer is affected by clinical factors such as stage and grade. Therefore, we wanted to combine the RNA network and clinical factors to establish a comprehensive prognostic risk model. We performed multiple regression analyses, and the results indicated that age, histologic grade, and risk score were independent risk factors for shorter survival (Fig. 4C). Subsequently, we built a new prognostic model for predicting OS based on age, histologic grade, and the network risk score (Fig. 4D). Elderly patients with pancreatic cancer with high histological grades and high-risk scores had worse prognoses. The ROC curve analysis revealed that the MIR600HG/hsa-miR-342-3p/ANLN-related risk model effectively predicted the 1-year, 3-year, and 5-year survival rates of patients with pancreatic cancer based on TCGA dataset (Fig. 4E). The calibration plot indicates the stability and reliability of the risk model (Fig. S5).

### Clinical relevance of the MIR600HG

The expression of MIR600HG in different tissues, T stage, histological grade, and M stage is shown in Fig. S6A–D. The expression of MIR600HG in tumor tissues was lower than that in normal tissues (Fig. S6A). The expression of MIR600HG in the T3&T4, G3&G4, and M1 subgroups was lower than that in the T1&T2, G1&G2, and M0 subgroups, respectively (Fig. S6B–D). Moreover, we performed a K–M survival analysis of MIR600HG expression in different T stages, and patients with pancreatic cancer presenting low expression of MIR600HG experienced a shorter OS (Fig. S6E–F). Then, we performed a pan-cancer analysis of the MIR600HG expression in 31 types of cancers (Fig. S6G). The results confirmed that MIR600HG was expressed at low levels in 23 types of tumors, including pancreatic cancer (*P* < 0.05).

### Clinical relevance of the ANLN

**Figure 5:**
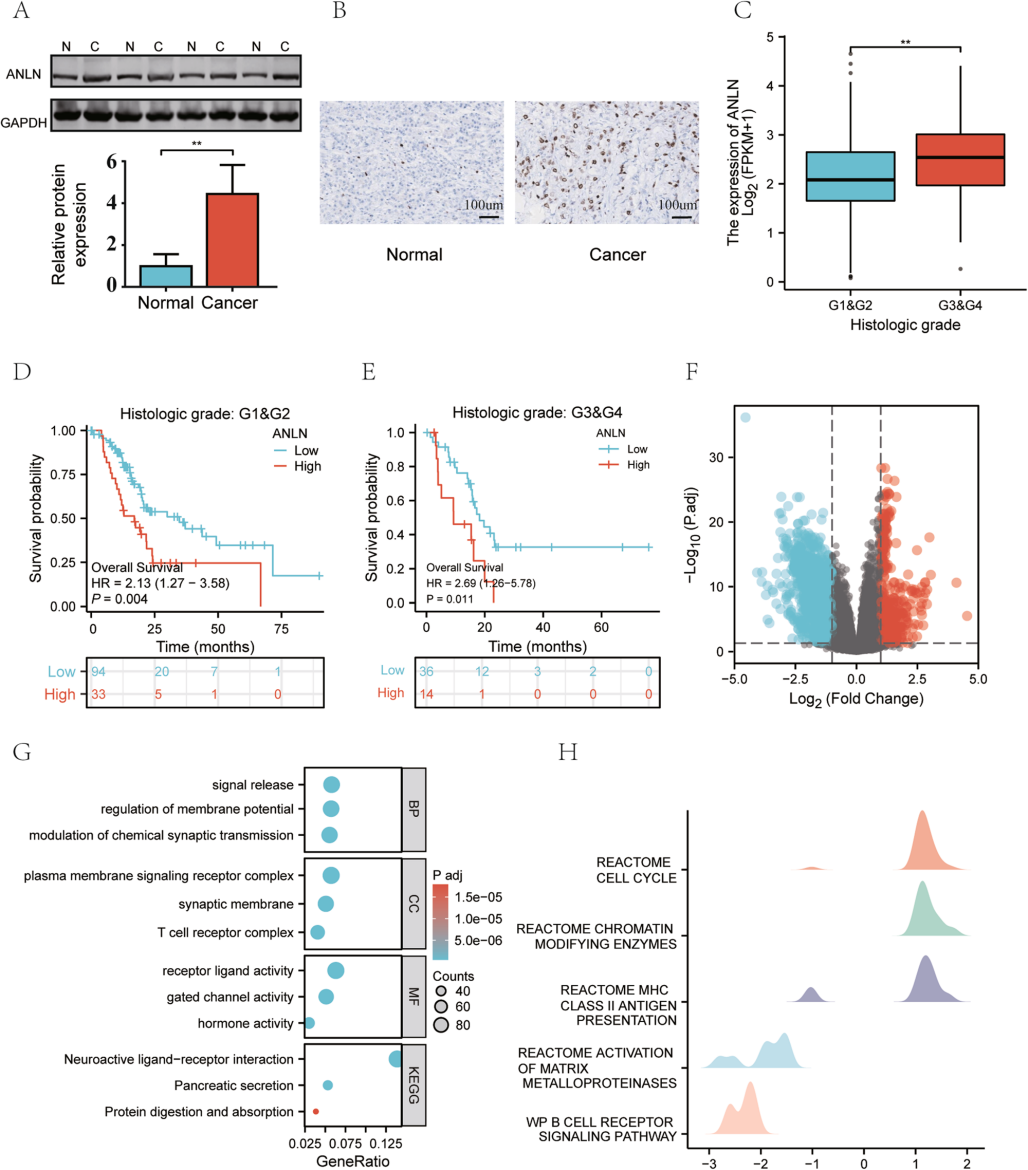
Relationship between ANLN expression and the clinical features of patients with pancreatic cancer. (**A**) Comparison of ANLN expression between normal tissue and pancreatic cancer tissue by WB, the original images of full-length blots was provided in supplementary Information file. (**B**) Comparison of the expression of the ANLN protein between the normal pancreas and pancreatic cancer by IHC. (**C**) The expression of ANLN in different histological grades of pancreatic cancer. (**D**–**E**) Kaplan‒Meier survival curves stratified by the high/low expression of ANLN in different histological grades of pancreatic cancer. (**F**) Volcano plot showing differentially expressed mRNAs between patients with high expression of ANLN and low expression of ANLN from TCGA. (**G**–**H**) GO enrichment analysis, KEGG enrichment analysis, and GSEA of differentially expressed mRNAs.

To explore the clinical value of ANLN, we first analyzed the expression of ANLN. As shown in Fig. 5A, the expression of ANLN in tumor tissues was higher than that in normal tissues. IHC confirmed the high expression of ANLN in pancreatic cancer (Fig. 5B). The expression of ANLN was further confirmed by using the TGCA and HPA database (Fig. S7A–B). We also found that the expression of ANLN in the G3&G4 subgroup was higher than that in the G1&G2 subgroup (Fig. 5C). However, ANLN expression was not significantly different among the pathological stage, T stage, N stage, and M stage subgroups (Fig. S7C–F). In addition, the K–M survival analysis revealed that patients with pancreatic cancer in different histologic grade subgroups presenting high ANLN expression experienced shorter OS (Fig. 5D–E).

Subsequently, patients were divided into an ANLN high expression group and an ANLN low expression group to explore the mechanism by which ANLN promotes cancer. We analyzed the DEGs between the two groups. The volcano plot showed 1667 genes with significant differences in expression (including 406 upregulated mRNAs and 1261 downregulated mRNAs) (|logFC|> 1 & p.adj < 0.05) (Fig. 5F). The GO/KEGG enrichment analyses of these 1667 genes are shown in Fig. 5G. In addition, the results of the GSEA were enriched in the reactome cell cycle, reactome chromatin-modifying enzymes, and reactome MHC class antigen presentation (Fig. 5H). MHC antigen presentation is an important part of the immune response, which suggests that ANLN may be related to the immune response.

### The relationship between the risk score of the RNA network and immune cell infiltration

**Figure 6:**
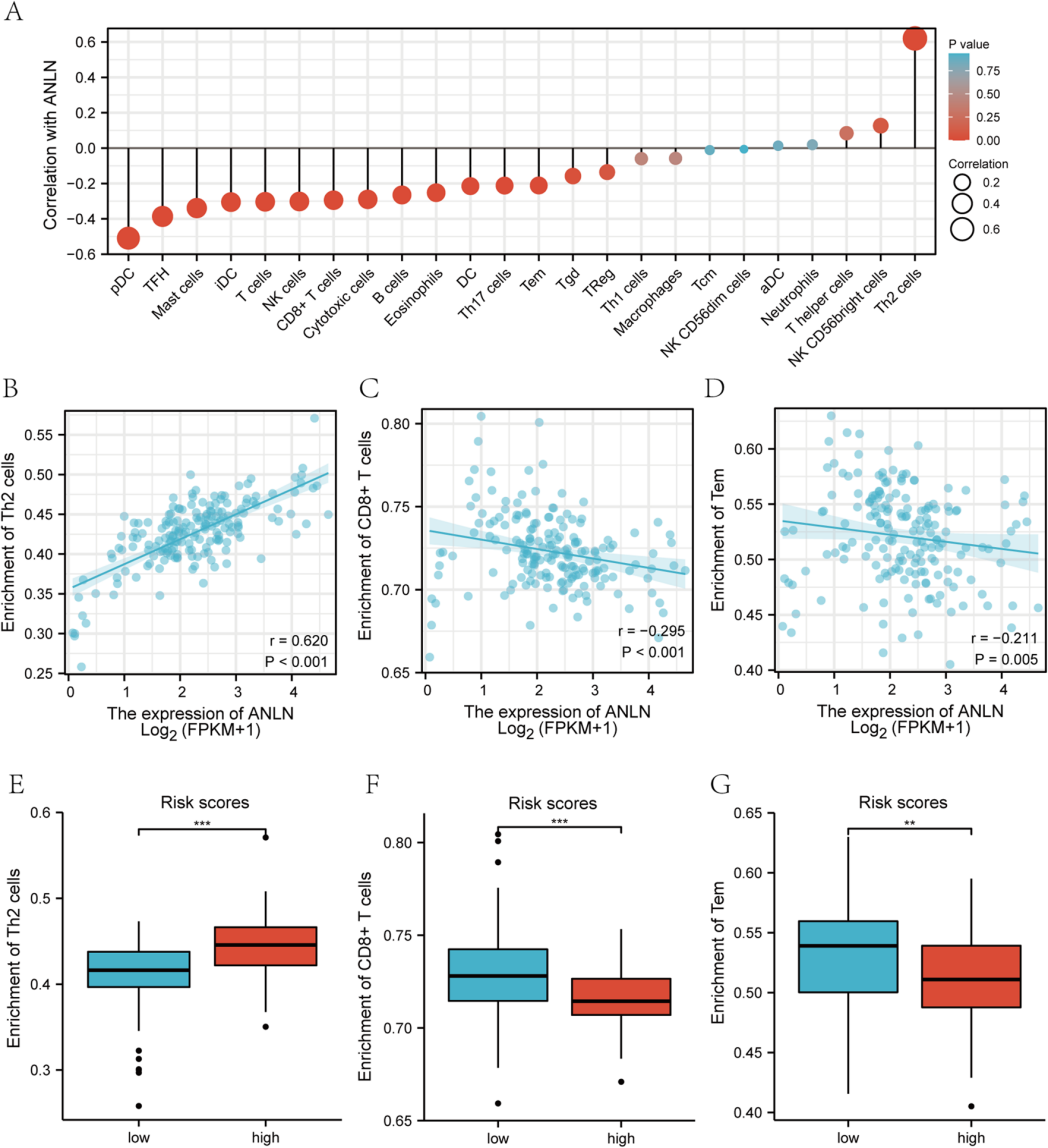
Relationship between ANLN expression and immune cell infiltration. (**A**) The correlation analysis between ANLN expression and infiltrating immune infiltration. (**B**–**D**) The scatterplot shows the correlations of ANLN expression with immune cells (Th2 cells, CD8 + T cells, and Tem cells). (**E**–**G**) The scatterplot shows the correlations of RNA network risk score with immune cells (Th2 cells, CD8 + T cells, and Tem cells).

We construct a risk score containing ANLN, previous studies^19,20^ have found that the expression of ANLN is closely related to immune infiltration, so we explored whether the risk score containing ANLN can affect immune infiltration of pancreatic cancer. We performed ssGSEA to calculate the immune infiltration score of 24 immune cells in each sample, and the Cox proportional hazard regression method was used to evaluate the relationship between the infiltration of 24 types of immune cells and survival. The results of the multivariate analysis suggested that the infiltration of 8 types of immune cells was an independent prognostic factor for pancreatic cancer (*P* < 0.05) (Table S3). To explore the effect of ANLN on different immune cells, we analyzed the correlation between ANLN expression and the infiltrating immune cells. As shown in Fig. 6A, three of the abovementioned 8 types of cells (Th2 cells, CD8 + T cells, and Tem cells) were significant in the Cox analysis and correlation analysis. The correlations between the infiltrating immune cells and ANLN expression are shown (Fig. 6B–D). As a result, Th2 cells were positively correlated with ANLN expression. In contrast, CD8 + T cells and Tem cells were negatively correlated with ANLN expression. Next, we analyzed the immune cell infiltration in different subgroups of the RNA network risk score. In the low-risk group, Th2 infiltration levels were higher, while CD8 + T cells and Tem cells infiltration levels were lower (Fig. 6E–G). Moreover, the results of the K-M survival analysis revealed that patients with high immune infiltration of Tem or Th2 cells experienced shorter OS, while the level of CD8 + T cell infiltration was not related to the OS of patients with pancreatic cancer (Fig. S8).

### ANLN may mediate immune cell infiltration by regulating chemokines

**Figure 7:**
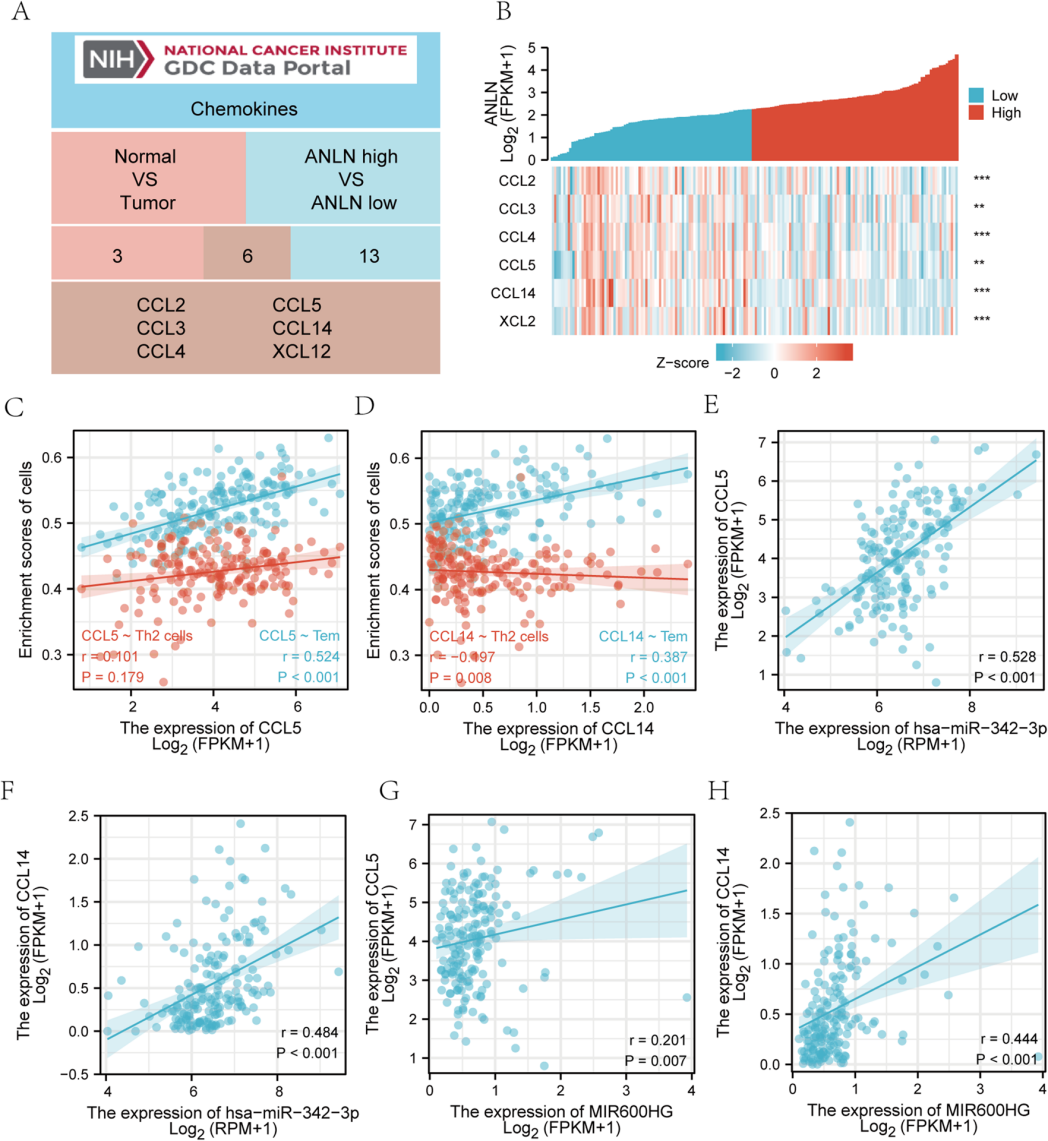
Relationship between ANLN and chemokines. (**A**) Venn diagram showing the significantly differentially expressed chemokines. (**B**) The heatmap shows the expression of chemokines in patients with different levels of ANLN expression. (**C**–**D**) The scatterplot shows the correlations between the expression of chemokines (CCL5 and CCL14) with immune cells (Th2 cells and Tem cells). (**E**–**F**) The scatterplot shows the correlations between the expression of chemokines (CCL5 and CCL14) with hsa-miR-342-3p expression. (**G**–**H**) The scatterplot shows the correlations between the expression of chemokines (CCL5 and CCL14) with MIR600HG expression.

It is well known that chemokines could regulate the infiltration of immune cells. Therefore, we intersected the differentially expressed chemokines in different tissues and the differentially expressed chemokines in different ANLN subgroups. As shown in Fig. 7A, 6 common chemokines (CCL2, CCL3, CCL4, CCL5, CCL14, and XCL12) were shared between the two groups. Then, we examined the chemokines in different ANLN subgroups. In the two subgroups stratified by ANLN expression, the expression levels of 6 chemokines were significantly different (Fig. 7B). By performing a K–M survival analysis, CCL5 and CCL14 are associated with survival in patients with pancreatic cancer (Fig. S9).

Next, we analyzed the relationship between the chemokines and immune cells. The results suggested that the expression of CCL5 was positively correlated with Tem cells (Fig. 7C). CCL14 was positively correlated with Tem cells but negatively correlated with Th2 cells (Fig. 7D). Next, we analyzed the correlation between the chemokines and the MIR600HG/hsa-miR-342-3p/ANLN network. The results showed that CCL5 and CCL14 were positively correlated with hsa-miR-342-3p and MIR600HG, respectively (Fig. 7E–H).

## Discussion

Pancreatic cancer is a highly invasive digestive tumor^1^. Although surgery is the only possible method to achieve a clinical cure for pancreatic cancer, a large number of patients have lost the chance to receive surgical treatment before they are diagnosed^21^. Due to the insensitivity of pancreatic cancer to radiotherapy and chemotherapy, immunotherapy and targeted therapy may be a new breakthrough in the treatment of pancreatic cancer^5^. However, accurate and effective therapeutic targets for pancreatic cancer are still lacking. The lncRNA‒miRNA–mRNA network has been confirmed to be involved in regulating various tumor immune microenvironments^22,23^. Therefore, we aim to establish a lncRNA‒miRNA–mRNA network related to immune cell infiltration and the prognosis and provide ideas for further explorations of biomarkers and potential therapeutic targets of pancreatic cancer.

Here, we screened key DEGs in pancreatic cancer, searched the upstream miRNAs and lncRNAs based on key DEGs, and finally constructed a MIR600HG/hsa-miR-342-3p/ANLN network (Fig. 3G). In addition, we constructed a prognostic risk model for pancreatic cancer based on the MIR600HG/hsa-miR-342-3p/ANLN network score. The ROC curve analysis showed that AUC values for 1-, 3-, and 5-year survival rates were 0.823, 0.843, and 0.862 (Fig. 4E), respectively. This finding indicates that the model effectively predicts the survival time of patients with pancreatic cancer. We also evaluated the relationship between the network score and immune cell infiltration. The results showed that ANLN expression was upregulated and that the RNA network score was higher in the high immune infiltration group.

ANLN, a gene that encodes an actin-binding protein, is a member of a cluster of proteins involved in mitosis/cytokinesis and is a part of the cleavage groove^24^. Studies have found that ANLN is involved in the occurrence and development of tumor cells^25,26^. Here, we observed high expression of ANLN in pancreatic cancer at the mRNA and protein levels, consistent with the results reported by Olakowski et al.^27^. By analyzing the clinical characteristics of patients with different ANLN expression levels, we found that ANLN is related to tumor differentiation in pancreatic cancer. The study by Wang et al.^28^ supported our conclusion, but they also observed differences in tumor size and TNM stage. This discrepancy may be because most patients diagnosed with pancreatic cancer were in the middle and late stages, and the number of patients in the early stage was insufficient. Furthermore, we performed an enrichment analysis of DEGs in patients with high and low ANLN expression, and the results showed that DEGs were enriched in the cell cycle, DNA repair, extracellular matrix, and other processes, which was mutually confirmed with our conclusions described above.

The upstream miRNA (hsa-miR-342-3p) and lncRNA (MIR600HG) of ANLN were identified among the DEGs in pancreatic cancer. Studies have shown that miR-342-3p is a tumor suppressor molecule. Overexpression of miR-342-3p in B-cell lymphoma downregulates E-cadherin, thereby inhibiting tumor progression^29^. E-cadherin is an important cell adhesion molecule. At same time, we found that DEGs between ANLN subgroups were enriched in cell adhesion functions, which is consistent with the function reported for miR-342-3p. This result further validates that hsa-miR-342-3p may act as a tumor suppressor molecule by inhibiting ANLN expression. Furthermore, Gao et al.^30^ found that miR-342-3p was correlated with the tumor grade in gliomas, and we obtained the same results for patients with pancreatic cancer.

Next, we identified the upstream lncRNA (MIR600HG) of hsa-miR-342-3p and observed significantly altered expression of MIR600HG in pancreatic cancer. Consistent with a previous study^31,32^, we found that MIR600HG was associated with survival outcomes of patients with pancreatic cancer. Yao et al. showed that MIR600HG inhibited tumor invasion and enhanced chemical sensitivity by targeting ALDH1A3 in colorectal cancer^33^. In oropharyngeal squamous cell carcinoma, MIR600HG affects the occurrence and development of tumors through autophagy-related pathways^34^.

According to previous reports^35^, miRNAs regulate the expression of mRNAs. We found that hsa-miR-342-3p was negatively correlated with ANLN expression, potentially because hsa-miR-342-3p forms complementary base pairs with the 3'-UTR of ANLN to exert its inhibitory effect. As shown in the study by Liu et al.^36^, miR-342-3p directly targets the 3'-UTR of IGF-1R, inhibiting the expression of IGF-1R. The online database miRWalk verified our hypothesis that a binding site for miR-342-3p is located in the 3'-UTR of ANLN, and this result may be further verified by performing a luciferase reporter assay. Compared with miRNAs, the regulatory mechanisms of lncRNAs are more complex and diverse^37^, including the premise of miRNAs, competitive combinations, and regulation of transcription factor activity. Competing endogenous RNAs (ceRNAs) are one of the regulatory mechanisms with more reports^38^. LncRNAs competitively bind miRNAs to relieve the inhibitory effect of miRNAs on mRNAs^38^. However, unlike the ceRNA regulation model, we found that MIR600HG was positively correlated with hsa-miR-342-3p, suggesting that MIR600HG was not associated with competitive inhibition of hsa-miR-342-3p expression. Previous studies^39^ have shown that lncRNAs regulate downstream mRNAs either by adsorbing microRNAs or regulating the transcription level of miRNA precursors. In addition, lncRNAs may be directly used as precursors to produce miRNAs, and a positive correlation was observed between the expression of lncRNAs and miRNAs, while the expression of the target mRNAs were negatively correlated with miRNA expression in the sequencing or microarray results^40^. Previous studies^41^ have also found that lncRNAs bind transcription factors and localize to the promoter region of genes to promote gene transcription and lncRNA-mediated methylation of histones. Here, we speculate that MIR600HG may be a precursor transcriptional regulator of hsa-miR-342-3p, and the specific mechanism must be verified by conducting more experiments.

It is well known that immune cell infiltration plays an important role in the processes of tumor development, tumor immune monitoring, and tumor immune escape. Different infiltrating immune cell subsets in the pancreatic cancer microenvironment are considered characteristic and independent prognostic factors^42^. Consistently, we also found that immune cell infiltration was significantly associated with OS in patients with pancreatic cancer^43^. We found that ANLN expression was significantly associated with a variety of immune cells (Th2 cells, Tem cells, etc.). Th2 cells have been reported to be associated with tumor invasion and metastasis and a poor prognosis^44^. Th2 cells are strongly associated with prognosis of patients with pancreatic cancer^45^. Pancreatic cancer tissues with a high proportion of Th2 cells tend to be rich in fibrous components and M2 macrophages, which are all reported to exert cancer-promoting effects^46^. In addition, the Th2-induced tumor immune microenvironment is an important cause of insensitivity to chemotherapy and radiotherapy in patients with pancreatic cancer^47,48^. The imbalance of the Th1/Th2 ratio and the dominance of Th2 cells in pancreatic cancer may be the mechanism of tumor immune escape^49^. Memory T cells are divided into different subsets according to their phenotype and function, including central memory T cells (Tcm) and Tem cells. Tem cells are an important part of the immune defense and antigen clearance system in the body, which produces rapid and efficient immune responses against foreign antigens. Previous studies^50^ have mainly focused on infectious diseases such as tuberculosis and viruses. Recently, it has been reported^51^ that Tem cells have great potential in tumor immunotherapy and prognosis. Takahashi et al.^51^ reported that the Tem high infiltration group was predicted to have longer OS among patients with head and neck squamous cell carcinoma. Consistently, we found that Tem cells were associated with the prognosis of patients with pancreatic cancer. In addition, Tem cells are involved in tumor immunotherapy. In patients with breast cancer, Tem cells participate in PD1 immunotherapy and correlate with the heterogeneity in the treatment response to anti-PD1^52^. CD137 agonist-based combination immunotherapy enhances the activation of Tem cells and prolongs the survival of patients with pancreatic adenocarcinoma^53^. At the same time, great progress has been achieved in Tem cell-based tumor therapy. A nanovaccine increased the number of Tem cells in mice, which resulted in long-term immune memory that recognized and killed cancer cells^54^. Tumor-infiltrating lymphocyte (TIL) therapy exerts strong curative effects on the treatment of solid tumors. TILs are often dominated by Tem cells, which also suggests the potential role of Tem cells in tumor treatment^55^. Here, we identified correlations between ANLN expression and the numbers of Th2 and Tem cells, suggesting that ANLN may participate in the immune process of pancreatic cancer through Th2 and Tem cells.

Chemokines are chemoattractant cytokines that play a key role in regulating the migration and infiltration of immune cell populations. In pancreatic cancer, the levels of many chemokines are significantly changed and are closely related to the immune cell infiltration and prognosis of patients^56^. We obtained ANLN-related chemokines by comparing ANLN high expression subgroups with low expression subgroups. By comparing the differentially expressed chemokines, CCL5 and CCL14 were identified. Here, we found that changes in the expression of CL5 and CCL14 were consistent in pancreatic cancer, while they had opposite prognostic effects. CCL5 is associated with a poor prognosis of cancer^57–59^, while the effect of CCL14 on the prognosis was inconsistent^60–62^. Furthermore, we found that this phenomenon also occurred in colorectal cancer and other tumors in TCGA database. This result may be because ANLN is a common upstream regulatory molecule of CCL5 and CCL14, but the biological functions of CCL5 and CCL14 are different. This finding requires further verification by detecting the expression of CCL5 and CCL14 after regulating ANLN expression. In addition, the high-affinity receptor for CCL5 is chemokine receptor 5 (CCR5), while the high-affinity receptor for CCL14 is chemokine receptor 1 (CCR1)^63^. This difference in receptors may also explain why these chemokines have different functions. Interestingly, we found that CCR5 was one of the main chemokine receptors expressed on the Tem cell surface, which suggests that ANLN may affect Tem cell infiltration through the CCL5-CCR5 pathway.

This study systematically analyzed multiple pancreatic cancer datasets, but some limitations to this study should be noted. First, the functions of MIR600HG, hsa-miR-342-3p and ANLN in pancreatic cancer require further experimental research. Then, the relationships between the RNA network and chemokines and immune cells must be further verified in vitro and in vivo. In addition, the sample size must be expanded to verify the sensitivity and specificity of the risk prognostic model based on the MIR600HG/hsa-miR-342-3p/ANLN network.

## Conclusion

In conclusion, we identified the MIR600HG/hsa-miR-342-3p/ANLN network in pancreatic cancer by performing a comprehensive analysis. The risk prognostic model constructed based on the RNA network effectively predicts the prognosis of patients with pancreatic cancer. Furthermore, we explored the relationship between the RNA network and the immune microenvironment and preliminarily explained the relationship between the RNA network and immune cell infiltration in pancreatic cancer. This study not only provides a new model for evaluating the prognosis of patients with pancreatic cancer but also identifies a potential target for clinical immunotherapy.

## Materials and methods

### RNA-seq data

Three pancreatic cancer microarrays datasets (GSE15471, GSE16515, and GSE46234) were obtained using the GEOquery package (2.54.1). GSE15471 included 39 tumor tissues and 38 nontumor tissues. GSE16515 included 36 tumor tissues and 16 nontumor tissues. GSE46234 included 4 tumor tissues and 4 nontumor tissues. TCGA datasets including 178 tumor tissues and 4 nontumor tissues were downloaded from the TCGA database using TCGAbiolinks package in R (version 3.6.3).

### Screening of DEGs and enrichment analysis

DEGs were obtained using the limma package (3.42.2). DEGs satisfying |logFC|> 1 and *p*.adj < 0.05 in the three microarray datasets were selected. Then, we used the ggplot2 package (3.3.3) to draw a Venn diagram and identify the common DEGs. TCGA data were used for verification. The enrichment analysis was performed using the clusterProfiler package (3.14.3). The Database for Annotation, Visualization, and Integrated Discovery (https://david.ncifcrf.gov/) was used to conduct Gene Ontology (GO) functional annotation and Kyoto Encyclopedia of Genes and Genomes (KEGG)^64–66^ pathway enrichment analyses.

### Screening of hub genes

The PPI network was constructed separately for DEGs using the STRING database (https://string-db.org/). Then, the PPI network was further screened using cytoHubba in Cytoscape, and the top 10 genes (upregulated DEGs and downregulated DEGs) were extracted as hub genes according to the algorithm (Maximal Clique Centrality, MCC).

### Survival analysis

The surv_cutpoint function in the survminer package is used to calculate the cut-off value. survival regression was fitted using the survival package (3.3.1), and the results were visualized using the survminer package and the ggplot2 package.

### Identification of the upstream miRNA

Upstream miRNAs were predicted by the miRWalk online database (http://mirwalk.umm.uni-heidelberg.de/). The differentially expressed miRNAs in GSE163031, which included 12 tumor tissues and 3 nontumor tissues, were screened using R software. The common miRNAs of the upstream miRNAs and differentially expressed miRNAs were used in subsequent research.

### Identification of the upstream lncRNAs

Upstream lncRNAs of the miRNAs were predicted by the lncBase online database (https://dianalab.e-ce.uth.gr/html/diana/web/index.php). The differentially expressed lncRNAs were screened in TCGA dataset using R software. The common lncRNAs of the upstream lncRNAs and differentially expressed lncRNAs were used in subsequent research. The binding sites of lncRNAs and miRNAs were predicted using the lncBase online database.

### Construction of the lncRNA–miRNA–mRNA network prognostic model

A standard Cox proportional hazards model implemented in the R package survival (3.2–10) was used to construct the risk score basing the lncRNA–miRNA–mRNA network. The timeROC package (0.4) and the ggplot2 package (3.3.3) were utilized to draw time-dependent ROC curves. The area under curve (AUC) was calculated to compare the predictive ability. We developed a prognostic factor-based risk stratification nomogram for 1, 3-year overall survival with Cox proportional hazards regression analysis using the rms (6.2-0) package.

### Relationship between the RNA network and clinical characteristics and the pan-cancer analysis

According to the TCGA database, the relationship between the expression of components of the RNA network (MIR600HG/hsa-miR-342-3p/ANLN) and different clinical characteristics was detected using R software, and the ggplot2 package was used for data visualization. Pan-cancer analysis was completed based on the TCGA database using R software. Images of immunohistochemical staining for ANLN in tumor tissues and pancreatic cancer tissues were obtained from the HPA website (https://www.proteinatlas.org/).

### Western blot

Western blot was performed according to previous studies^67^. In brief, the extracted tissues were denatured by electrophoresis and then transferred to a polyvinylidene fluoride membrane (Merck Millipore Ltd., Germany). The membrane was blocked with 5% skim milk and incubated with primary antibodies at 4 °C overnight. Finally, the membrane was incubated with IRDye 800CW secondary antibodies (LI-COR, USA) (1:10,000) at room temperature for 1 h, and the proteins were visualized and analyzed with Odyssey® Imaging System (LI-COR, USA). Primary antibodies against the following target proteins were used: ANLN (Santa, 1:500) and GAPDH (Sigma, 1:8000).

### Immunohistochemistry

As previously reported^68^**,** Tissues were fixed in 4% paraformaldehyde, paraffin embedded and sectioned. The ANLN antibody (Santa, 1:50) was incubated overnight, the secondary antibody was incubated at 37 °C for half an hour. The sections were observed under light microscope after adding chromogenic agent.

### Immune infiltrate levels and RNA network expression analysis

The marker genes of 28 immune cell types were obtained for the ssGSEA. The infiltration level was quantified using the GSVA package (1.34.0). A Cox multivariate model was used to screen significant immune cells and thereby construct an immune infiltration score. The correlation between immune cell infiltration and the RNA network was analyzed using R software.

### Screening of chemokines

Differentially expressed chemokines were obtained between normal tissues and pancreatic cancer tissues, and then the chemokines that changed in the differentially expressed ANLN subgroup were obtained. Finally, the common chemokines between the two groups were obtained using R software.

### Statistical analysis

The statistical analysis was performed using the bioinformatic tools mentioned above. A *P* value < 0.05 was considered statistically significant.

### Ethics approval and consent to participate

This study was approved by the Ethical Committee of the First Affiliated Hospital of Harbin Medical University (IRB-AF/SC-04/02.0).

### Supplementary Information

Supplementary Information 1.

Supplementary Information 2.

## Data Availability

This study conduct analysis based on TCGA public database (https://gdc.xenahubs.net) and GEO public database (https://www.ncbi.nlm.nih.gov/gds/). All data generated or analyzed during this study are included in this article.
