# Supplementary material for: Identification of MIR600HG/hsa-miR-342-3p/ANLN network as a potential prognosis biomarker associated with lmmune infiltrates in pancreatic cancer
Source: Sci Rep. 2023 Sep 23;13:15919. doi: 10.1038/s41598-023-43174-y (PMC10517933; doi:10.1038/s41598-023-43174-y)
Supplement: Supplementary file 1 — Supplementary Information 1. [file 41598_2023_43174_MOESM1_ESM.docx]

Supplementary figure


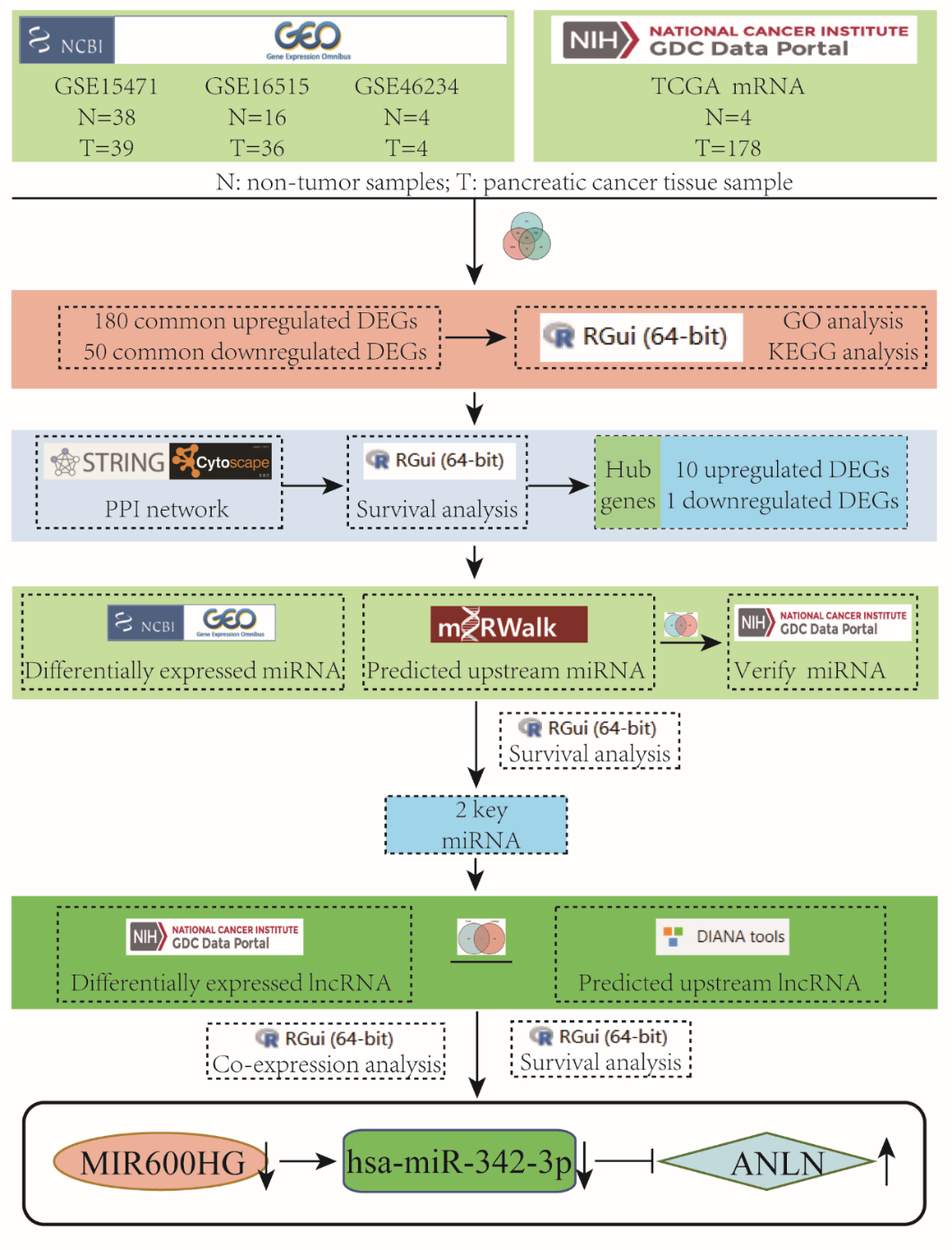


Figure S1. Flowchart of construction and analysis of the RNAs network


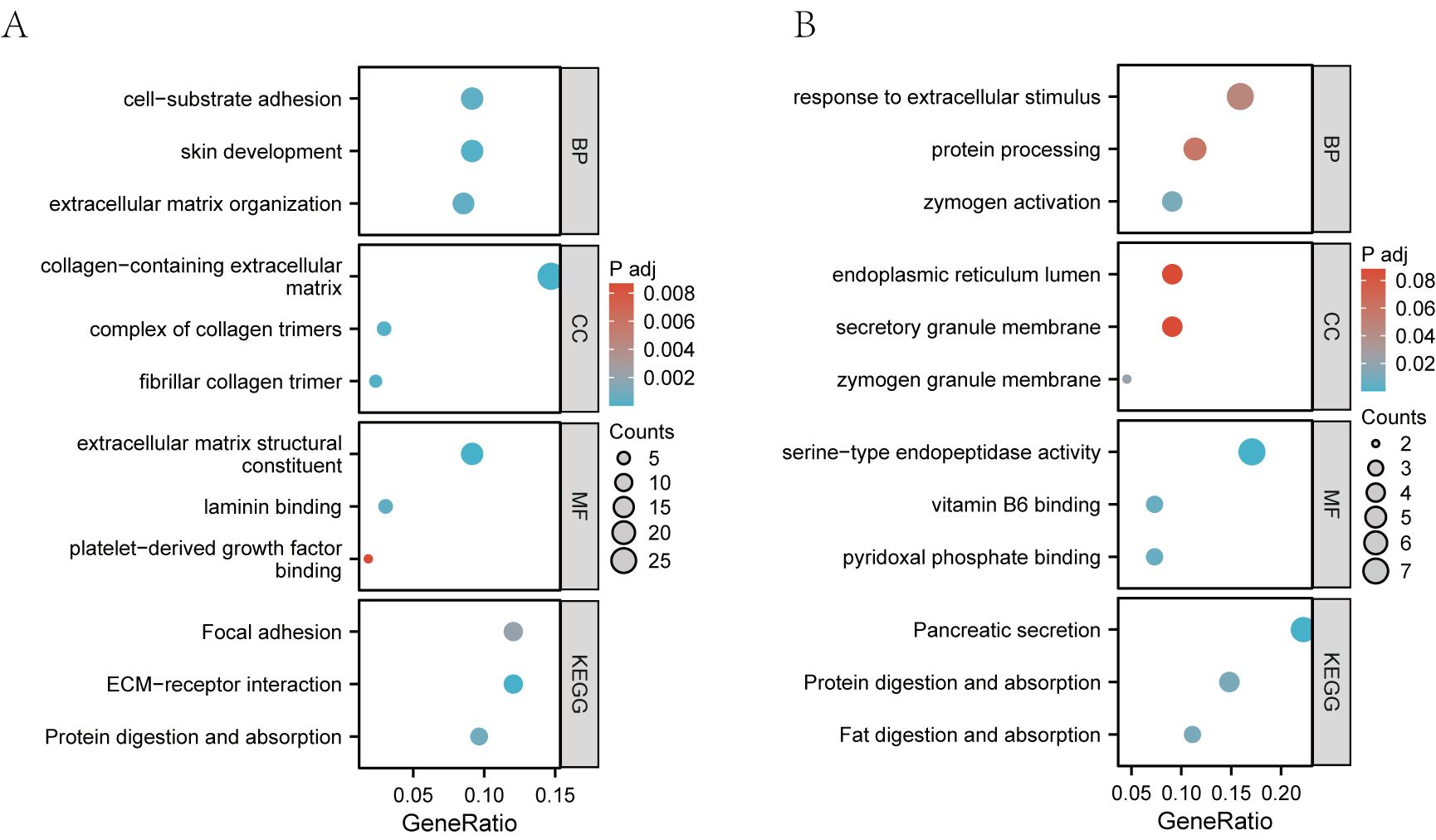


Figure S2. GO and KEGG pathway enrichment analysis of DEGs. (A) Up-regulated DEGs. (B) Down-regulated DEGs.


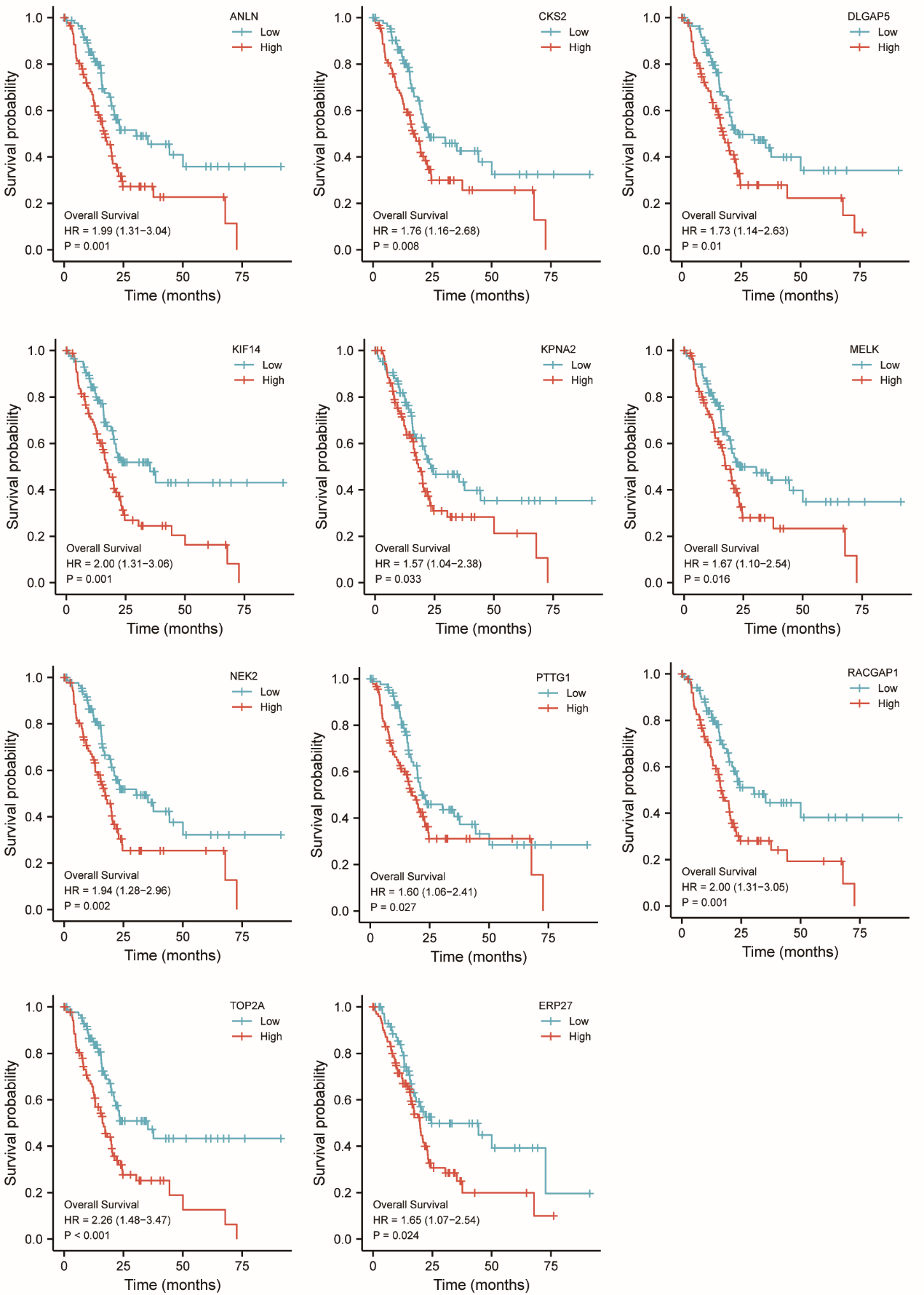


Figure S3. Kaplan-Meier survival curve by the hub genes.


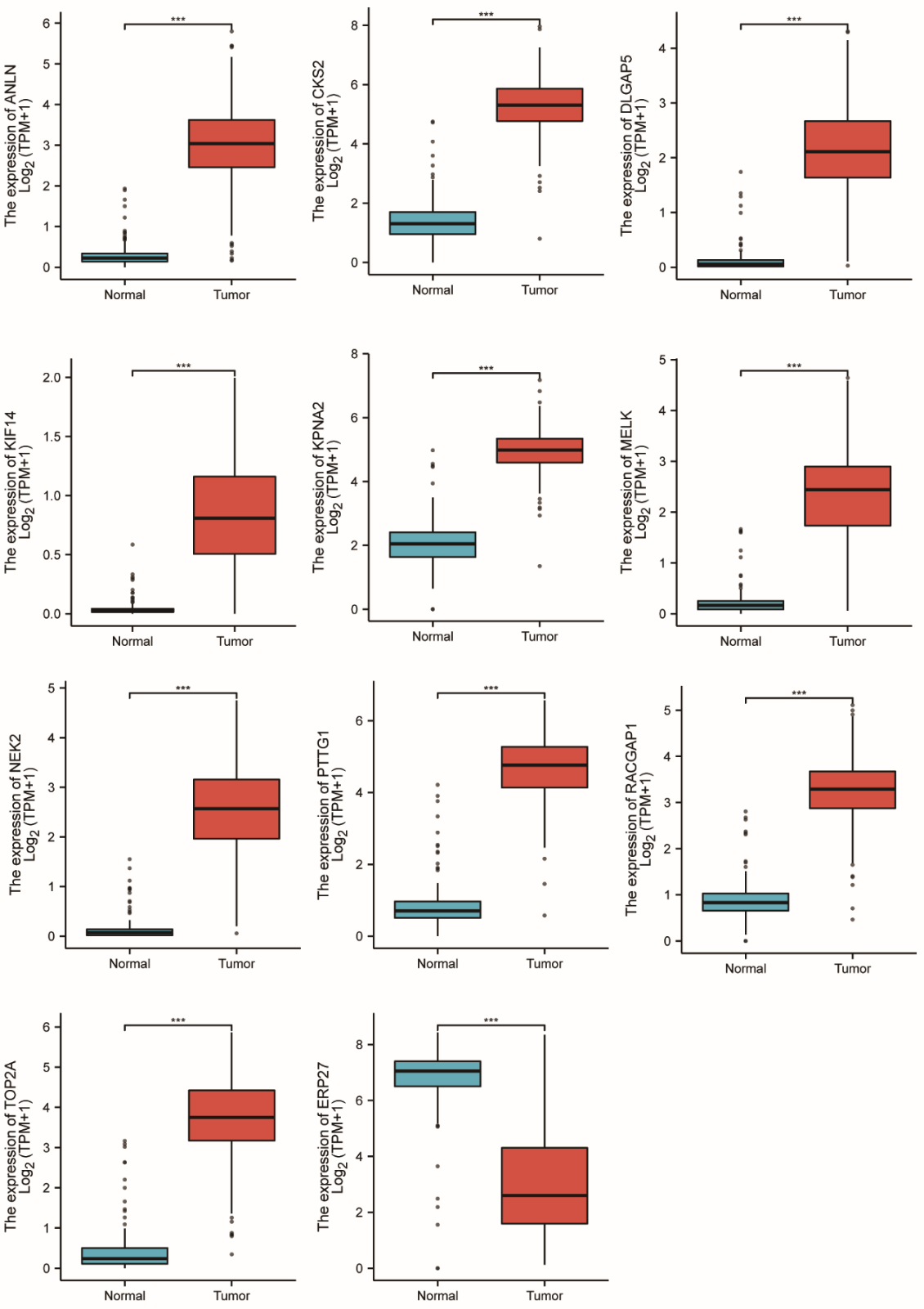


Figure S4. The expression of the hub genes between the pancreas and pancreatic cancer.


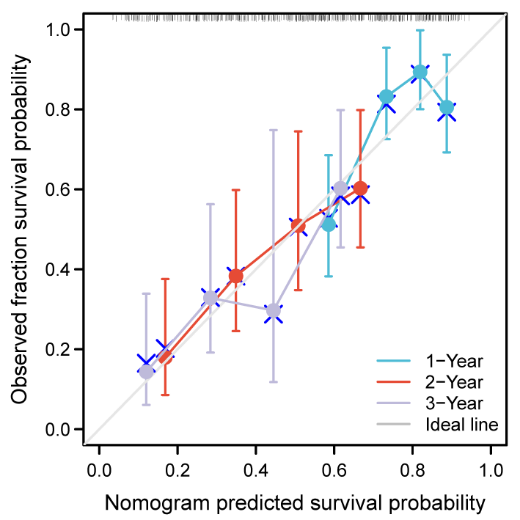


Figure S5. Calibration plot of the MIR600HG/hsa-miR-342-3p/ANLN related risk model


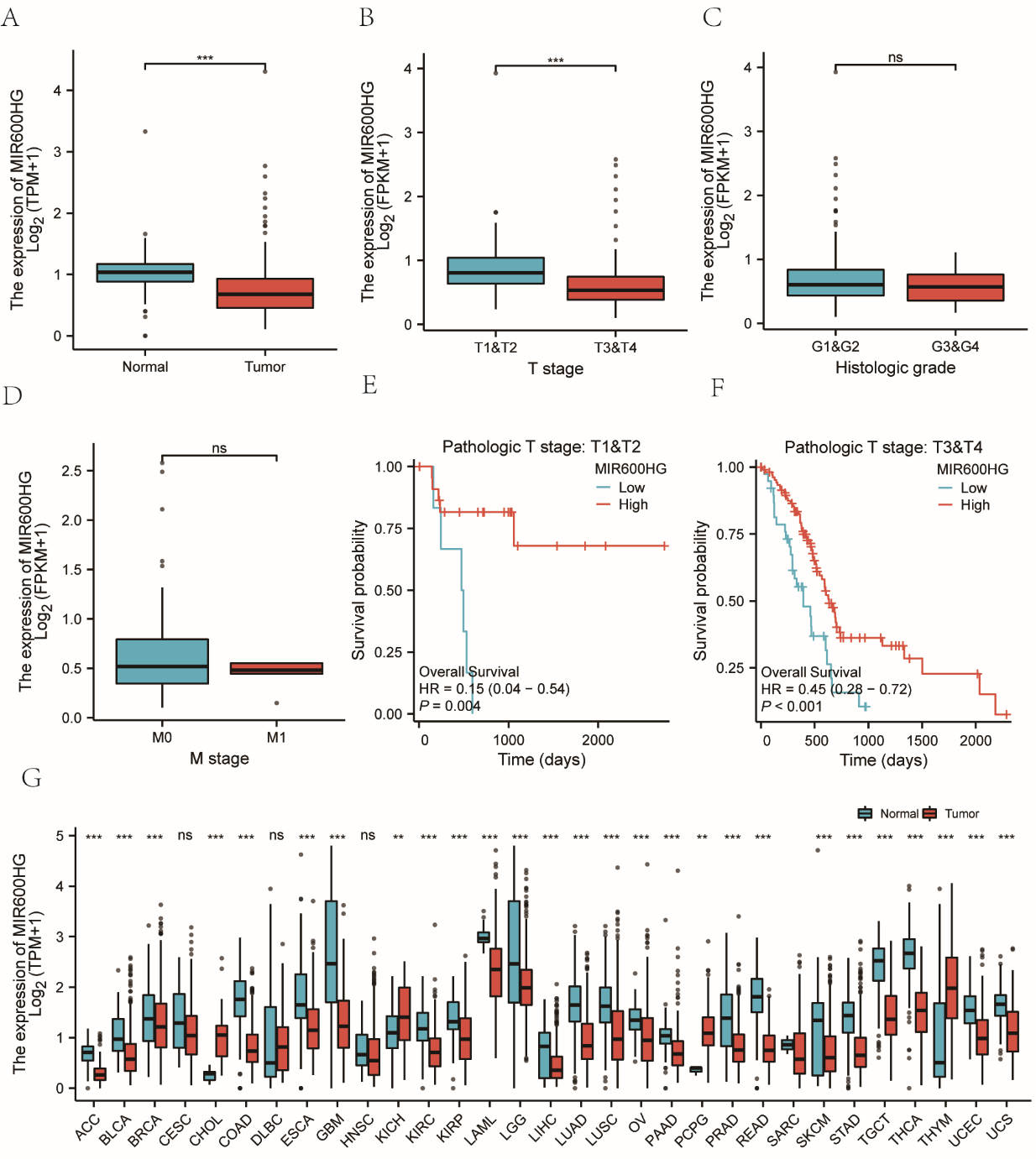


Figure S6. Relationship between MIR600HG and clinical features. (A) The expression. of MIR600HG between. tumor tissues and pancreatic cancer tissues. (B-D) The expression of MIR600HG in different T stage, histological. grade, and M stage of pancreatic cancer. (E-F) KAPLAN-MEIER survival curves. stratified by the high/low expression of MIR600HG in different T stages. (G) The pan-cancer. analysis of the expression of MIR600HG based on TCGA dataset.


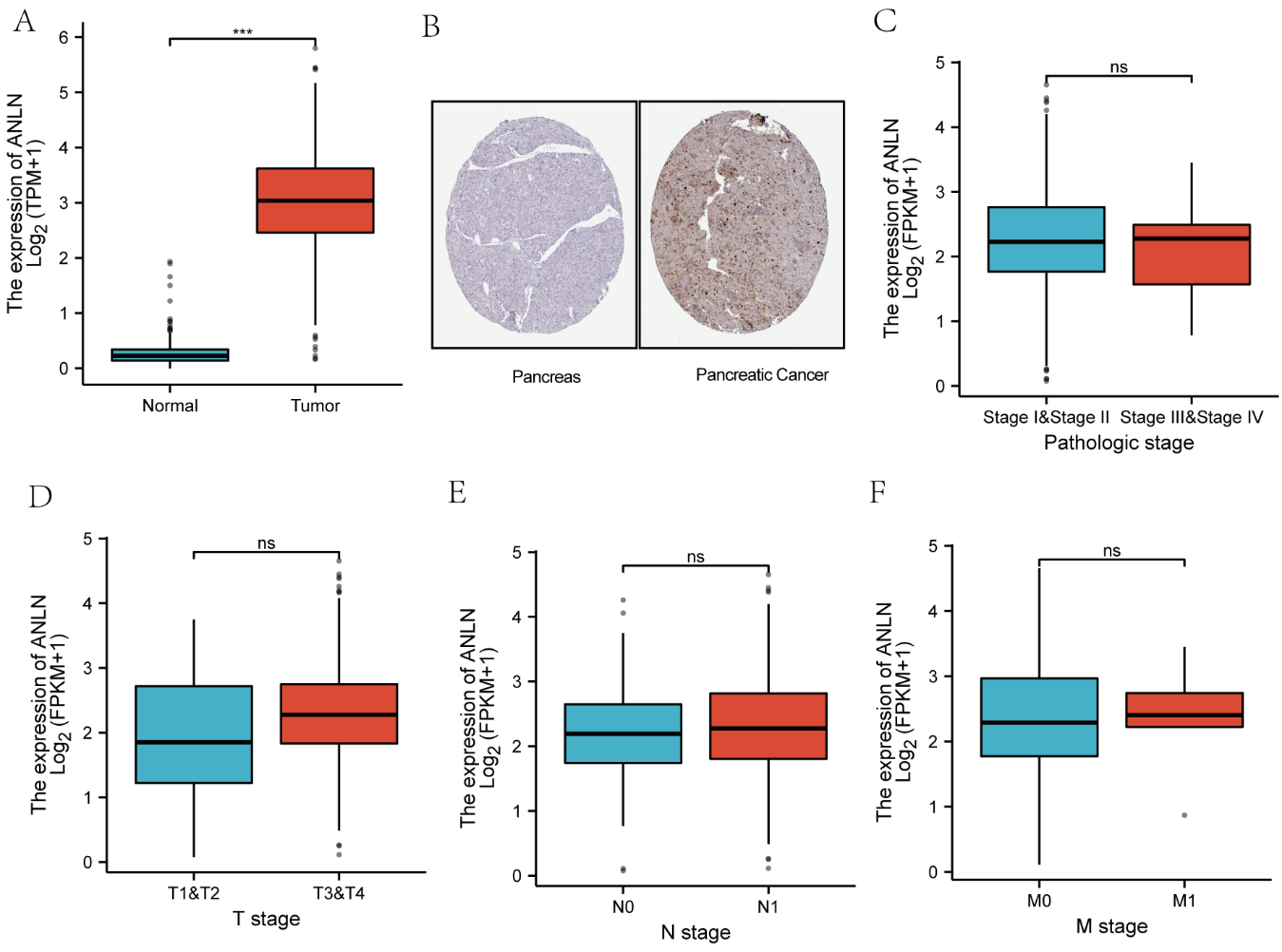


Figure S7. Relationship between the expression of ANLN and different clinical stages. (A) The expression of ANLN in pancreatic cancer tissues and normal tissues was detected from TCGA. (B) The expression of ANLN in pancreatic cancer tissues and adjacent normal tissues was detected from HPA. (C) Pathologic stage. (D) T stage. (E) N stage. (F) M stage.


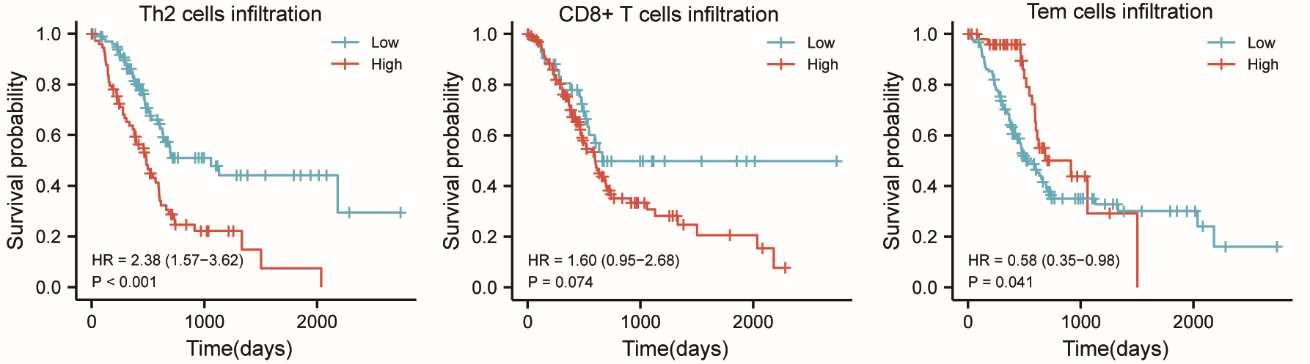


Figure S8. Kaplan-Meier survival curve by the immune cells (Th2 cell, CD8+T cell, Tem cell).


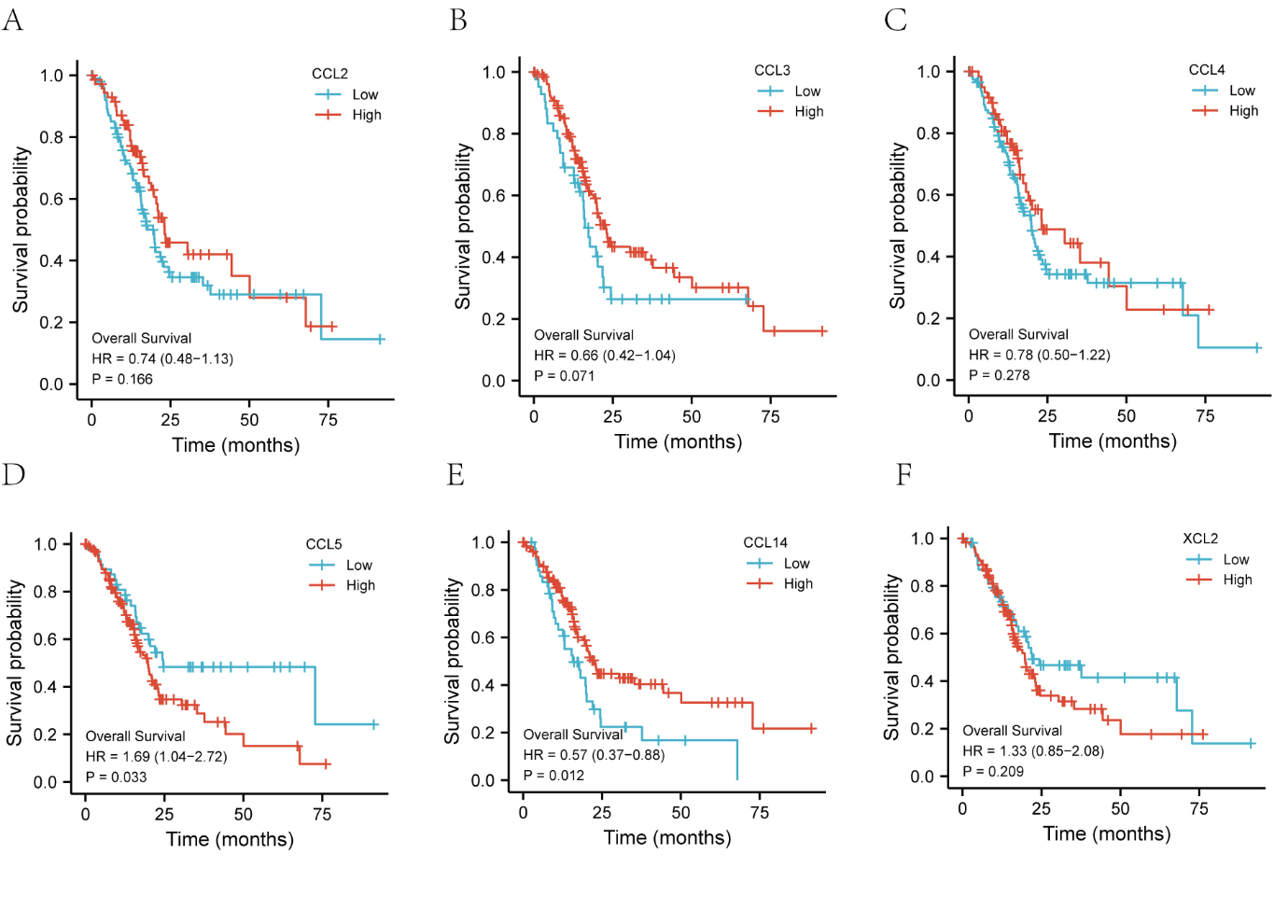


Figure S9. Kaplan-Meier survival curve by the chemokines (CCL2, CCL3, CCL4, CCL5, CCL14 and XCL12)

**Supplementary table**

Table S1. The expression. correlation between the miRNA and mRNA.

| miRNA | mRNA | R | P |
| --- | --- | --- | --- |
| hsa-miR-342-3p | ANLN | −0.251 | **< 0.001** |
| hsa-miR-371a-5p | CKS2 | −0.082 | 0.280 |
| hsa-miR-216a-3p | ERP27 | 0.721 | **< 0.001** |
| hsa-miR-128-3p | ERP27 | −0.159 | **0.034** |
| hsa-miR-3921 | ERP27 | −0.037 | 0.623 |
| hsa-miR-148a-3p | KIF14 | −0.200 | **0.008** |
| hsa-miR-181d-5p | MELK | −0.088 | 0.241 |
| hsa-miR-181b-5p | MELK | 0.298 | **< 0.001** |
| hsa-miR-6807-5p | NEK2 | 0.033 | 0.664 |
| hsa-miR-3120-3p | NEK2 | NA | NA |
| hsa-miR-1233-5p | NEK2 | NA | NA |
| hsa-miR-485-5p | NEK2 | −0.203 | **0.007** |
| hsa-miR-6785-5p | NEK2 | −0.024 | 0.756 |
| hsa-miR-331-3p | RACGAP1 | 0.169 | **0.024** |
| hsa-miR-4689 | RACGAP1 | −0.152 | **0.043** |
| hsa-miR-4695-5p | RACGAP1 | NA | NA |
| hsa-miR-6858-5p | RACGAP1 | 0.090 | 0.236 |
| hsa-miR-6891-5p | TOP2A | 0.032 | 0.675 |

Table S2. Correlation of gene. expression (MIR600HG, hsa-miR-342-3p, and ANLN) with clinicopathological. variables in pancreatic cancer of TCGA datasets

| **Characteristic** | **ANLN** | | | | **hsa-miR-342-3p** | | | | **MIR600HG** | | |
| --- | --- | --- | --- | --- | --- | --- | --- | --- | --- | --- | --- |
|  | **Low** | **High** | | ***p*** | | **Low** | **High** | ***p*** | **Low** | **High** | ***p*** |
| n | 89 | 89 | |  | | 89 | 90 |  | 89 | 89 |  |
| **Gender** |  |  | | 0.292 | |  |  | 0.325 |  |  | 0.651 |
| Female | 44 | 36 | |  | | 36 | 44 |  | 42 | 38 |  |
| Male | 45 | 53 | |  | | 53 | 46 |  | 47 | 51 |  |
| **Age** |  |  | | 0.764 | |  |  | 0.943 |  |  | 0.368 |
| <=65 | 45 | 48 | |  | | 46 | 48 |  | 43 | 50 |  |
| >65 | 44 | 41 | |  | | 43 | 42 |  | 46 | 39 |  |
| **T stage** |  |  | | 0.729 | |  |  | **0.045** |  |  | **0.001** |
| T1 | 3 | 4 | |  | | 4 | 3 |  | 1 | 6 |  |
| T2 | 14 | 10 | |  | | 6 | 18 |  | 6 | 18 |  |
| T3 | 68 | 74 | |  | | 77 | 66 |  | 79 | 63 |  |
| T4 | 2 | 1 | |  | | 1 | 2 |  | 3 | 0 |  |
| **N stage** |  |  | | 0.754 | |  |  | 1.000 |  |  | 1.000 |
| N0 | 26 | 24 | |  | | 25 | 25 |  | 25 | 25 |  |
| N1 | 59 | 64 | |  | | 61 | 63 |  | 63 | 60 |  |
| **M stage** |  |  | | 1.000 | |  |  | **0.026** |  |  | 0.068 |
| M0 | 37 | 42 | |  | | 37 | 43 |  | 43 | 36 |  |
| M1 | 2 | 3 | |  | | 5 | 0 |  | 5 | 0 |  |
| **Pathologic stage** |  |  | | 0.758 | |  |  | **0.039** |  |  | **0.001** |
| Stage I | 12 | 9 | |  | | 7 | 14 |  | 5 | 16 |  |
| Stage II | 71 | 75 | |  | | 74 | 73 |  | 75 | 71 |  |
| Stage III | 2 | 1 | |  | | 1 | 2 |  | 3 | 0 |  |
| Stage IV | 2 | 3 | |  | | 5 | 0 |  | 5 | 0 |  |
| **Histologic grade** |  |  | **< 0.001** | | |  |  | 0.989 |  |  | 0.581 |
| G1 | 26 | 5 | |  | | 15 | 16 |  | 12 | 19 |  |
| G2 | 46 | 49 | |  | | 48 | 48 |  | 50 | 45 |  |
| G3 | 15 | 33 | |  | | 23 | 25 |  | 25 | 23 |  |
| G4 | 1 | 1 | |  | | 1 | 1 |  | 1 | 1 |  |
| **Smoker** |  |  | | 0.235 | |  |  | 0.605 |  |  | 1.000 |
| No | 37 | 28 | |  | | 33 | 33 |  | 33 | 32 |  |
| Yes | 36 | 43 | |  | | 35 | 44 |  | 40 | 39 |  |
| **Alcohol** |  |  | | 0.447 | |  |  | 0.207 |  |  | 1.000 |
| No | 35 | 30 | |  | | 36 | 29 |  | 32 | 33 |  |
| Yes | 47 | 54 | |  | | 45 | 57 |  | 50 | 51 |  |
| **diabetes** |  |  | | 0.187 | |  |  | 0.208 |  |  | 0.994 |
| No | 50 | 58 | |  | | 55 | 54 |  | 52 | 56 |  |
| Yes | 23 | 15 | |  | | 14 | 24 |  | 19 | 19 |  |
| **chronic pancreatitis** |  |  | | **0.021** | |  |  | 0.748 |  |  | 1.000 |
| No | 68 | 60 | |  | | 58 | 71 |  | 63 | 65 |  |
| Yes | 2 | 11 | |  | | 7 | 6 |  | 6 | 7 |  |

Table S3. COX multivariate. model was used to analyze the relationship. between immune cell infiltration and prognosis of patients with pancreatic cancer

| Characteristics | Total(N) | Multivariate analysis | |
| --- | --- | --- | --- |
|  |  | Hazard ratio (95% CI) | P value |
| aDC | 178 | 84.203 (4.065-1744.146) | **0.004** |
| B cells | 178 | 0.741 (0.016-33.832) | 0.878 |
| CD8 T cells | 178 | 11166836318064.283 (12653.588-9854773030615273963520.000) | **0.004** |
| Cytotoxic cells | 178 | 18.137 (0.015-22502.279) | 0.425 |
| DC | 178 | 57.531 (0.403-8213.571) | 0.109 |
| Eosinophils | 178 | 0.001 (0.000-603.137) | 0.300 |
| iDC | 178 | 0.576 (0.000-55385.369) | 0.925 |
| Macrophages | 178 | 843.297 (1.677-423952.215) | **0.034** |
| Mast cells | 178 | 20.331 (0.050-8247.502) | 0.326 |
| Neutrophils | 178 | 0.170 (0.001-33.514) | 0.511 |
| NK CD56bright cells | 178 | 0.657 (0.002-239.003) | 0.889 |
| NK CD56dim cells | 178 | 2.100 (0.006-707.169) | 0.803 |
| NK cells | 178 | 1.327 (0.000-256631.658) | 0.964 |
| pDC | 178 | 2.645 (0.055-127.412) | 0.623 |
| T cells | 178 | 0.030 (0.000-28.849) | 0.317 |
| T helper cells | 178 | 0.000 (0.000-0.000) | **0.002** |
| Tcm | 178 | 5294458.788 (510.457-54914097425.988) | **0.001** |
| Tem | 178 | 0.000 (0.000-0.000) | **<0.001** |
| TFH | 178 | 0.106 (0.000-1424.964) | 0.643 |
| Tgd | 178 | 0.001 (0.000-4.584) | 0.111 |
| Th1 cells | 178 | 0.604 (0.000-22671.257) | 0.925 |
| Th17 cells | 178 | 7.449 (0.639-86.834) | 0.109 |
| Th2 cells | 178 | 4539557442079.214 (93167144.472-221189367633732320.000) | **<0.001** |
| TReg | 178 | 0.003 (0.000-0.073) | **<0.001** |
