# Supplementary figures and images for: Identification of MIR600HG/hsa-miR-342-3p/ANLN network as a potential prognosis biomarker associated with lmmune infiltrates in pancreatic cancer
Source: Sci Rep. 2023 Sep 23;13:15919. doi: 10.1038/s41598-023-43174-y (PMC10517933; doi:10.1038/s41598-023-43174-y)

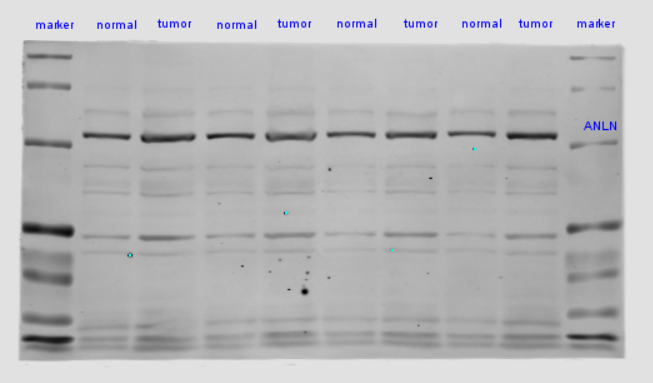


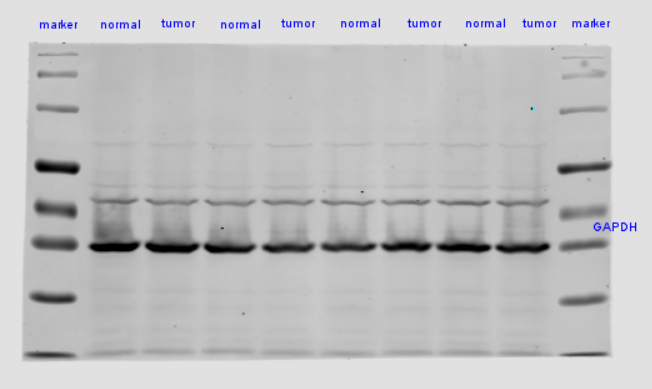

Supplement: Supplementary file 2 — Supplementary Information 2. [file 41598_2023_43174_MOESM2_ESM.docx]
